# Synthesis of ZnO Nanostructures for Low Temperature CO and UV Sensing

**DOI:** 10.3390/s121013842

**Published:** 2012-10-16

**Authors:** Muhammad Amin, Umair Manzoor, Mohammad Islam, Arshad Saleem Bhatti, Nazar Abbas Shah

**Affiliations:** 1 Thin Films Technology Laboratory, Department of Physics, COMSATS Institute of Information Technology, Islamabad 44000, Pakistan; E-Mails: aminislamabad@yahoo.com (M.A.); nazar_abbas@comsats.edu.pk (N.A.S.); 2 Alamoudi Water Chair, King Saud University, P.O. Box 2460, Riyadh 11451, Saudi Arabia; 3 Centre for Micro & Nano Devices, Department of Physics, COMSATS Institute of Information Technology, Islamabad 44000, Pakistan; E-Mail: asbhatti@comsats.edu.pk; 4 Center of Excellence for Research in Engineering Materials (CEREM), King Saud University, P.O. Box 800, Riyadh 11421, Saudi Arabia; E-Mail: miqureshi@ksu.edu.sa

**Keywords:** ZnO nanostructures, gas sensor, CO sensor, UV sensor, optical properties

## Abstract

In this paper, synthesis and results of the low temperature sensing of carbon monoxide (CO) gas and room temperature UV sensors using one dimensional (1-D) ZnO nanostructures are presented. Comb-like structures, belts and rods, and needle-shaped nanobelts were synthesized by varying synthesis temperature using a vapor transport method. Needle-like ZnO nanobelts are unique as, according to our knowledge, there is no evidence of such morphology in previous literature. The structural, morphological and optical characterization was carried out using X-ray diffraction, scanning electron microscopy and diffused reflectance spectroscopy techniques. It was observed that the sensing response of comb-like structures for UV light was greater as compared to the other grown structures. Comb-like structure based gas sensors successfully detect CO at 75 °C while other structures did not show any response.

## 1. Introduction

Oxides are the basis of various smart and functional materials [1]. Device fabrication using oxide semiconductors has gained significant importance, because physical properties of these oxides can be tuned. Among oxide semiconductors, zinc oxide (ZnO) has wide band gap energy (3.37 eV at room temperature), large exciton binding energy (60 meV), and strong piezoelectric and optical properties.

One dimensional (1-D) ZnO nanostructures can be synthesized by numerous synthesis methods such as electrochemical, vapor phase, and liquid phase techniques [2–6]. The vapor transport method is one of the most extensively explored due to single crystal structure and high aspect ratio of the resulting nanostructures. The growth kinetics of ZnO nanostructures by the vapor liquid solid (VLS) method depends on many factors *i.e.*, activation energy of crystallization, radius of catalyst droplets, super-saturation, synthesis temperature, volume of liquid droplets, and the equilibrium vapor pressure of the system. The thermodynamic limit for the critical radius (*R_min_*) of the metal liquid cluster at high temperature is given by Equation (1) [6]:

(1) $$R_{\min}=\frac{2r_{LV}\alpha }{kT\ln S}$$

where *r_LV_* is the liquid vapor surface free energy, *α* is the molar volume of liquid and *S* is the vapor phase super-saturation. The above relation suggests that there is a critical limit on the diameter and the ratio of supersaturation whereas temperature has direct effect on the growth of 1-D structures.

Toxic gases can affect human life and health even at levels of few parts per million and therefore, highly sensitive gas sensors are needed. ZnO is a potential optical and gas sensor material due to its high sensitivity to toxic and combustible gases, carrier mobility, and good chemical and thermal stability at moderately high temperatures [7]. Recently it was reported that response time of ZnO based sensors strongly depends on the size, specific surface area and morphology [8]. The sensing mechanism of ZnO is linked with surface-controlled reactions in which grain size, defects, and oxygen adsorption play a crucial role in the sensing response [9].

In the present study, systematic control of different ZnO nanostructural morphologies and their optical and sensing properties are presented. These ZnO nanostructures are promising materials for low-temperature, low-cost and high-performance gas sensors. The results show good response to 200 ppm CO gas at low operating temperatures. The sensing properties of ZnO comb-like structures (without doping or surface modification) are unique as, to our knowledge, previously reported sensors all detect CO at relatively high temperatures.

## 2. Experimental Section

Source materials, zinc oxide (99.9%) and graphite in powder form, were mixed in 1:1 weight ratio for 2 hr in a planetary ball mill (Fritsch, Pulverisette 5). About 1 g of this mixture was transferred to an Al_2_O_3_ boat. Silicon substrates were placed on top of the boat for the deposition of ZnO nanostructures. The boat was placed in the center of a quartz tube (ϕ; 3.5 cm and length 100 cm). This quartz tube was then placed inside a tube furnace which was ramped to the required temperature (900–950 °C) at 10 °C/min. Argon was used as the carrier gas, while growth time and Ar flow rate were fixed at 60 min and 50 sccm, respectively, in all the experiments. The other end of quartz tube was attached to a flexible tube (ϕ; 1.0 cm and length 100 cm) and was kept open.

X-Ray diffraction (XRD) and scanning electron microscopy (SEM) were used to determine the crystal structure and morphology of the grown nanostructures. Bandgap energy was calculated using UV-VIS reflectance spectroscopy. The CO gas sensors were fabricated by depositing 50 nm thick gold electrodes onto quartz substrates through sputter deposition technique and the respective ZnO nanostructures were placed between electrodes (through doctor blading). The response of the device was measured by observing the resistance behavior upon exposure of the device to CO gas. Thick film sensors were heat treated in air for 3 hours at 400 °C before performing the gas sensing experiments and experiments were performed at 75 °C with 5 min cycles of air and 200 ppm CO gas (balance air). A multimeter (Keithly 2000) was used to measure the respective resistance values in air (R_a_) and the gas (R_g_).

## 3. Results and Discussion

**Figure 1.**
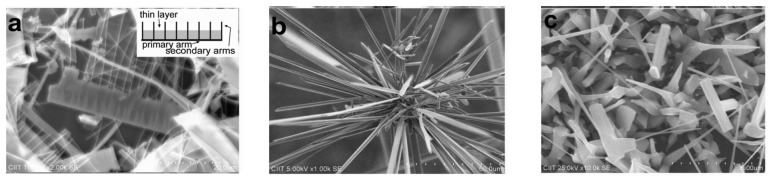
SEM images of different morphologies of ZnO Nanostructures at different synthesis temperatures. (**a**) Comb-like structures at 950 °C. The inset shows the schematic diagram of these combs; (**b**) Nanobelts with needles at 925 °C; (**c**) Mixture of rod and belts at 900 °C.

SEM micrographs of ZnO nanostructures synthesized at different temperatures are shown in Figure 1(a–c).

At 950 °C high density comb-like structures were synthesized, as shown in Figure 1(a). The general morphology of the samples consists of one-sided comb-shaped structures. Secondary arms of these comb-shaped dendrites are parallel to one another and their length may vary slightly from arm to arm. These secondary arms are connected from one side to the nanosheet to form the comb-like structure. Close examination suggests that most of the comb-like structures don't have a clear primary arm (main stem) and it seems that the secondary arms are placed parallel to each other and a thin layer is formed on only one side of the rods, giving it the comb-shape morphology [10]. A low magnification SEM image suggests that these combs are in large quantity on the substrate (Supporting Figure S1(a)). The average secondary arm diameter is 342 nm ± 26 nm and the average secondary arm length is 26 μm ± 15 μm. The inset in Figure 1(a) shows a schematic diagram of these comb-like structures.

Figure 1(b) shows a mixture of needle-shaped nanobelts grown from a single point at 925 °C. The length of these belts was found to be in tens of microns range and the average width of 36.2 μm ± 8.7 μm. These belts have faceted edges with needle-like ends. A low magnification SEM image is shown in Figure S1(c). At 900 °C, mixtures of nanobelts with long needle-like structures and rods were obtained (Figure 1(c)). These belts also have faceted edges having average tip width and thickness (from center of rod) of 189 nm ± 56 nm and 98 nm ± 43 nm, respectively. These structures are unique as, according to our knowledge, such type of ZnO nanostructures have not been reported in the literature.

All the SEM micrographs clearly suggest formation of different types of belt-shaped structures at all synthesis temperatures. The possible reason for this type of growth is attributed to higher supersaturation, surface instability and quick availability of ZnO polar surfaces, since at higher supersaturation, the atoms are readily available to these polar surfaces for random growth [5]. The possible reason of growth of secondary arms in case of comb-like structures can be linked to a self-catalyzed process for secondary arms and morphological instability in a supersaturated environment [11]. One of the possible reasons for the growth of needle-like nanobelts is that rates of crystal growth in different directions in ZnO are different *i.e.*, V<**011̄0**> > V<**011̄1**> > V<**0001**> [12] and due to this difference, the crystal growth face takes a specific shape. However, a detailed HRTEM analysis may be helpful to completely understand the growth kinetics of these belts. Overall, at different temperatures, the supersaturation conditions are different which ultimately changes the morphology.

**Figure 2.**
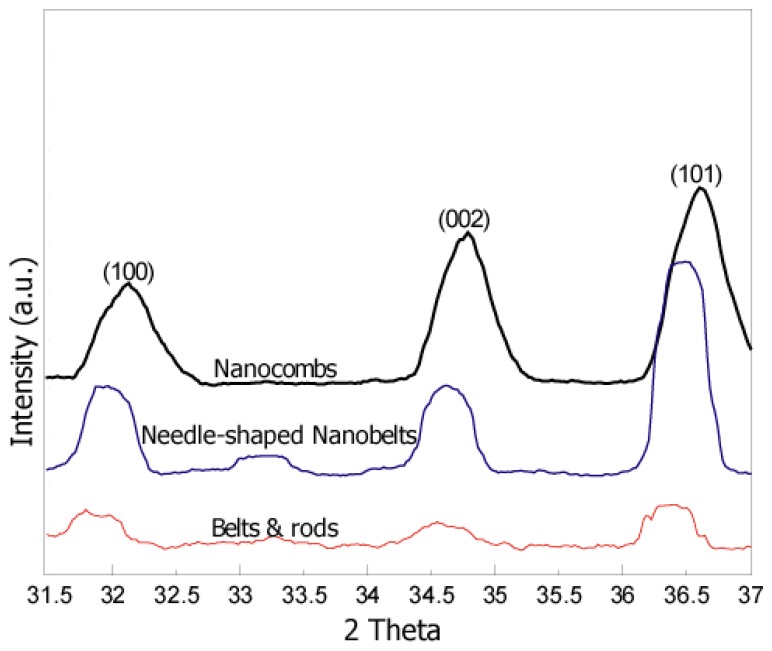
XRD results of as-synthesized ZnO nanostructures. Continuous shift in the peak towards higher angle and narrower FWHM suggests better crystalinity.

X-ray diffraction results (Figure 2) show presence of the main peaks of the ZnO structure. The figure clearly suggests that there is a systematic shift towards higher angle and full width at half-maximum (FWHM) is becoming narrower with an increase in the synthesis temperatures. The peak shift and narrow FWHM may be due to higher synthesis temperatures which help to enhance the mobility of atoms, subsequently resulting in reduced defect concentration and improved quality of the ZnO crystals [2,9]. Sharp and relatively high intensity peaks for comb-like structures (as compared to other structures) were observed which depicts high crystallinity of comb-like structures. Measured lattice parameters (a = b = 3.24 Å and c = 5.22 Å) for comb-like structures are comparable to the published values for the hexagonal Wurtzite structure.

**Figure 3.**
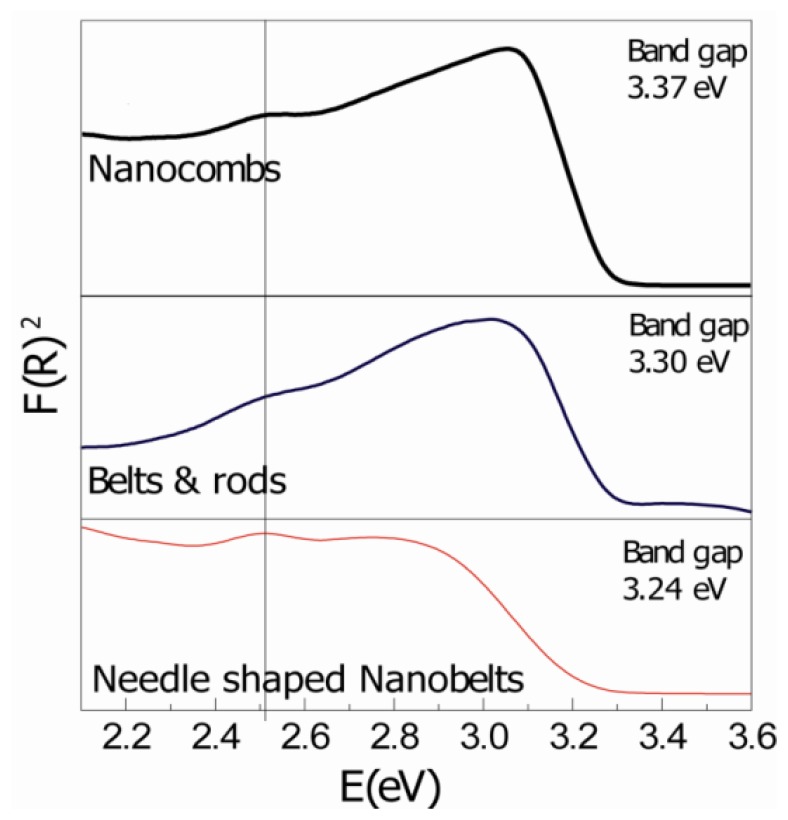
Reflectance Spectroscopy results after Kubelka-Munk treatment of as-synthesized ZnO nanostructures clearly shows direct band emission and a defect related smaller peak for all the samples. Comb-like structures show steeper slope as compared to other samples.

Room temperature UV-VIS reflectance spectra of as-grown samples, after Kubelka-Munk treatment, are shown in Figure 3. The spectrum of the as-synthesized ZnO nanostructures mainly consists of a strong UV peak and a weak defect related peak. The UV slope is the exciton recombination related near-band edge emission (NBE) of ZnO and the deep-level emission (DLE) usually results from radiative recombination of a photo-generated hole with an electron occupying the oxygen vacancy [13]. It was observed that with increase in synthesis temperature; there is (i) a systematic increase in peak intensity; (ii) steeper slope of the reflectance peak; and (iii) a clear shift in the peak towards lower wavelengths. There are many reasons for different peak intensities, *i.e.*, dispersed light due to scattering, electronic transitions in the samples, and variation in sample quantities. As it was not fixed, different nanostructured samples have different as-grown quantities on the substrate. However, increases in the intensity, steeper slopes, and shift in the slope with increase in the synthesis temperatures can be attributed to the improved quality of the crystal. Two different groups in independent studies concluded that after high temperature treatment, UV peak intensity increases significantly, indicating that quality of ZnO is improved through annealing [4,14]. Therefore, it can be deduced that high temperature synthesis plays an important role in tuning optical properties, and above-mentioned observations can be linked to the improvement in the crystal quality of ZnO comb-shape nanostructures. The calculated band gap values for all the structures are comparable to the theoretical value for ZnO.

The small defect related peak at 2.5 eV is apparent in all the samples. Point defects, *i.e.*, oxygen vacancy, interstitial oxygen, zinc vacancy, and impurities are considered to be possible origins for these bands [10,13,15]. However, its intensity is decreased for comb-like structures (high temperature synthesis). Point defects in ZnO, for entropy reasons, are thermodynamically stable at high temperatures [16]. Therefore it is difficult to completely remove the point defects and a minor defect related peak may always be present.

XRD and UV-VIS spectroscopy results are in perfect agreement with each other. Steeper slopes, and increased UV and XRD intensities suggest decreases in the crystal defects and improved quality of the ZnO. The XRD peak shift towards higher angle also indicates an improvement in the overall crystal structure. Hence, it can be suggested that high synthesis temperatures provide energy to ZnO atoms for enhance mobility and diffusion that could decrease defect density and improve the quality of ZnO comb-shape structures.

The sensing mechanism of ZnO based gas sensors relies on the change in electrical conductivity as a result of chemical reaction between gas molecules and the iono-sorbed oxygen on ZnO nanostructure surface. The relation for the change in electrical conductance is given by Equation (2) [17]:

(2) $$\Delta G=\frac{\left(\Delta n_{o}\left|e\right|\mu \pi r^{2}\right)}{l}$$

where ***Δ****n_o_* is the change in carrier's concentration; *e* is the electron charge, *μ* is the mobility of electrons, *r* is the radius, and *l* is length of the nanostructure channel. Thus the conductance of ZnO nanostructures will increase when 50 sccm (200 ppm) of CO gas is introduced into the sensor chamber due to the exchange of electrons between ionosorbed species and ZnO nanostructures. The chemical reaction between CO and ionic oxygen species is given by Equation (3):

(3) $$2CO+O_{2}^{-}\to 2CO_{2}+e^{-}$$

**Figure 4.**
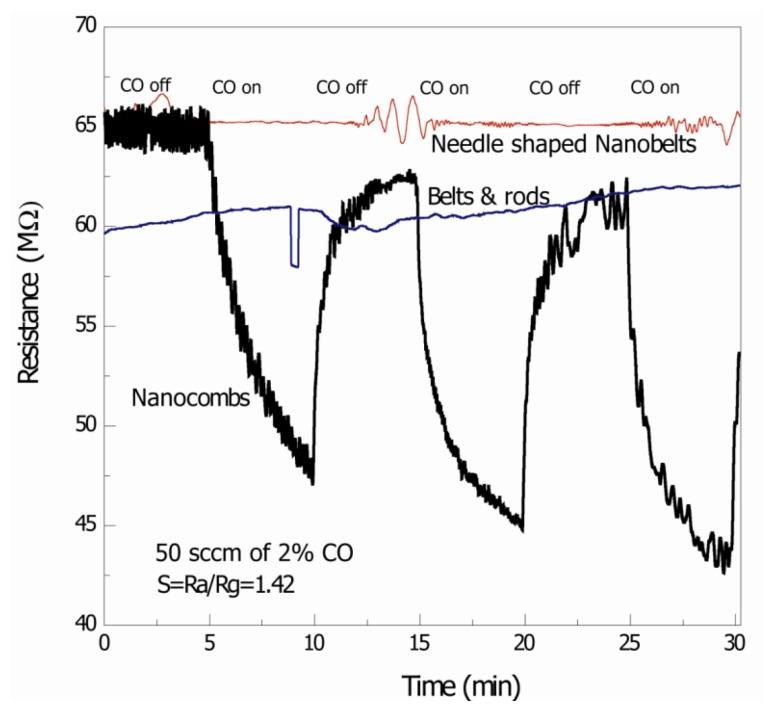
Sensing results for 2% CO gas at 75 °C suggest that only comb-like structures show cyclic changed in resistance with on and off cycles of CO gas.

Figure 4 is the signal response of the thick film ZnO nanostructure-based CO gas sensors, measured at 75 °C. It was observed that comb-like structures detect CO with response S = 1.42 (where S = R_a_/R_g_ where R_a_ and R_g_ are resistances in air and gas, respectively). On the other hand detection of CO with needle-shaped belts and mixture of belts and rods was negligible. The possible reasons for the detection of CO with comb-like structures may be the due to presence of non c-axis growth of secondary arms [8]. Different morphologies have different responses to the gas; as different crystallographic directions or atomic termination of the faces that are exposed to the gas are different [4]. The contribution of other crystallographic planes to the adsorption mechanisms may also be helpful towards improvement of sensing response of comb-like structures.

The specific surface area of ZnO nanostructures plays an important role in gas sensing. Qiu *et al.* fabricated a humidity sensor based on ZnO tetrapods and investigated the influence of the specific surface area on the response. They found that ZnO tetrapod films display much higher response to humidity than ZnO nanoparticles based films, and the response increases with decreasing tetrapod size, all of which is ascribed to the high specific surface area and high surface activity of the small tetrapod [18,19]. However, the BET analysis related to this study (not shown) showed that the specific surface area is comparable for all the structures and may not be a significant factor in the difference in response to CO gas. The other possibility, *i.e.*, better crystallinity of these structures, may not be a significant factor in detecting CO at low temperatures and many independent research groups have suggested only high temperature gas sensing even with good crystallinity of ZnO nanoparticles/nanostructures [20,21]. Response time of the comb-like structures is greater than recovery time which may be due to the slow surface adsorption of O_2_^−^·on ZnO. In a recent study, Lim *et al.* showed CO gas response of ZnO nanorods but the overall sensitivity was low and only occurred at high temperatures (300 °C) [22]. More recently, Zeng *et al.* [23] fabricated hierarchically porous ZnO nanosheet thin film based CO sensors with high responses and short recovery times, but again at high temperatures (300 °C). The response reported in this report is at much lower temperature and the comb-shape structures show better response as compared to other structures.

**Figure 5.**
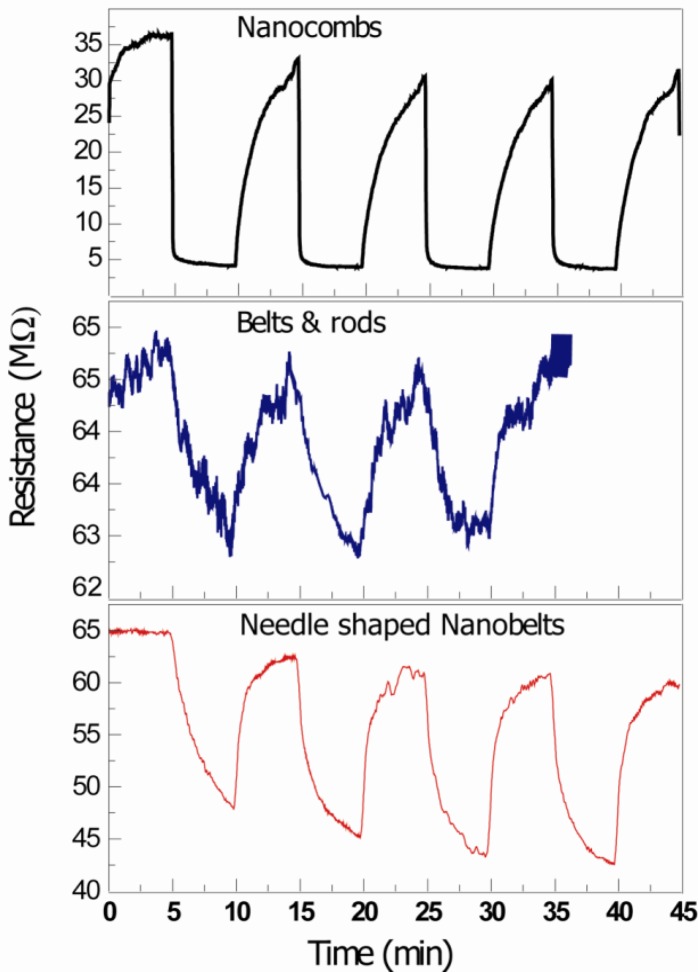
UV sensing at room temperature with (**a**) Comb-like structures; (**b**) Nanobelts with needles and (**c**) Mixture of rod and belts. 18 W UV lamp was used for this purpose.

Figure 5 shows the room temperature UV sensing results of ZnO nanostructures. The resistance decreases with exposure of UV light (wavelength ranges from 300 nm to 367 nm with 18 W UV Lamp, Phillips) and increases again when the UV lamp is switched off. When ZnO nanostructures are exposed to air, the negative space charge layer is created and the adsorbed oxygen molecule captures an electron from the conduction band (sensor exhibits higher resistivity). When the energy of photon is greater than the energy band gap E_g_, radiation is absorbed by the ZnO nanostructures based UV sensor, creating an electron-hole pair. The photo-generated, positively-charged hole neutralizes the chemisorbed oxygen responsible for the higher resistance, increasing the conductivity of the device. As a consequence, the conductivity in the material increases giving rise to photocurrent. This process goes on in a cyclic manner with the on-off switching of the UV light. It is expected that the reason for higher response time of ZnO comb-shape structures is not only due to better crystallinity (related to defects and bandgap energy) but also the non c-axis growth of secondary arms, as discussed above. The detailed HRTEM analysis of these comb-shaped structures have been reported in our previous articles [9,24].

A detailed summary of the results is given in Table 1. The table suggests that the results are systematic and comb-like structures not only have better crystallinity, but also better optical and sensing properties.

## 4. Conclusions

Different morphologies of ZnO nanostructures were successfully synthesized by a vapor transport method by carefully controlling the synthesis temperature. XRD and optical properties suggest good crystallinity with low defect concentrations. Gas (CO) sensing properties were investigated for the grown structures. Response of comb-like structures was found to be 1.4 at 75 °C, while belts and mixture of belts and rod like structures did not show any response. Comb-like structures also show much better response to UV light. High response of comb-like structures is attributed mainly to non c-axis growth.

## Supplementary Material



## Figures and Tables

**Table 1.** Detailed summary of the results.

**Morphology of Nanostructures**	**Synthesis Temperature (°C)**	**Band Gap by UV/Vis (eV)**	**Nanostructure Size (nm)**	**UV Sensing Response**	**CO Response (s)**
Comb-like structures	950	3.37	φ (secondary arms) = 242 nm	8.0	1.4
Belts with needle Like structures	925	3.30	Width = 2.5 μm	1.0	Negligible
Belts & rods	900	3.24	φ (tips) = 119 nm Width = 492 nm	1.4	Negligible
