# Supplementary material for: Synthesis of ZnO Nanostructures for Low Temperature CO and UV Sensing
Source: Sensors (Basel). 2012 Oct 16;12(10):13842–51. doi: 10.3390/s121013842 (PMC3545595; doi:10.3390/s121013842)
Supplement: Supplementary file 1 [file sensors-12-13842-s001.pdf]

OPEN ACCESS

sensors

ISSN 1424-8220
www.mdpi.com/journal/sensors

Supplementary Information

Synthesis of ZnO Nanostructures for Low Temperature CO and UV Sensing. *Sensors* 2012, *12*, 13842–13851

Muhammad Amin [1], Umair Manzoor [2,3,*], Mohammad Islam [4], Arshad Saleem Bhatti [3] and Nazar Abbas Shah [1]

1 Thin Films Technology Laboratory, Department of Physics, COMSATS Institute of Information Technology, Islamabad 44000, Pakistan; E-Mails: aminislamabad@yahoo.com (M.A.); nazar_abbas@comsats.edu.pk (N.A.S.)
2 Alamoudi Water Chair, King Saud University, P.O. Box 2460, Riyadh 11451, Saudi Arabia
3 Centre for Micro & Nano Devices, Department of Physics, COMSATS Institute of Information Technology, Islamabad 44000, Pakistan; E-Mail: asbhatti@comsats.edu.pk
4 Center of Excellence for Research in Engineering Materials (CEREM), King Saud University, P.O. Box 800, Riyadh 11421, Saudi Arabia; E-Mail: miqureshi@ksu.edu.sa

* Author to whom correspondence should be addressed; E-Mail: umanzoor@ksu.edu.sa; Tel.: +96-658-279-1919; Fax: +96-61-467-3739.

Figure S1. A Low magnification SEM images of (**a**) Comb-like structures (**b**) Nanobelts with needles.

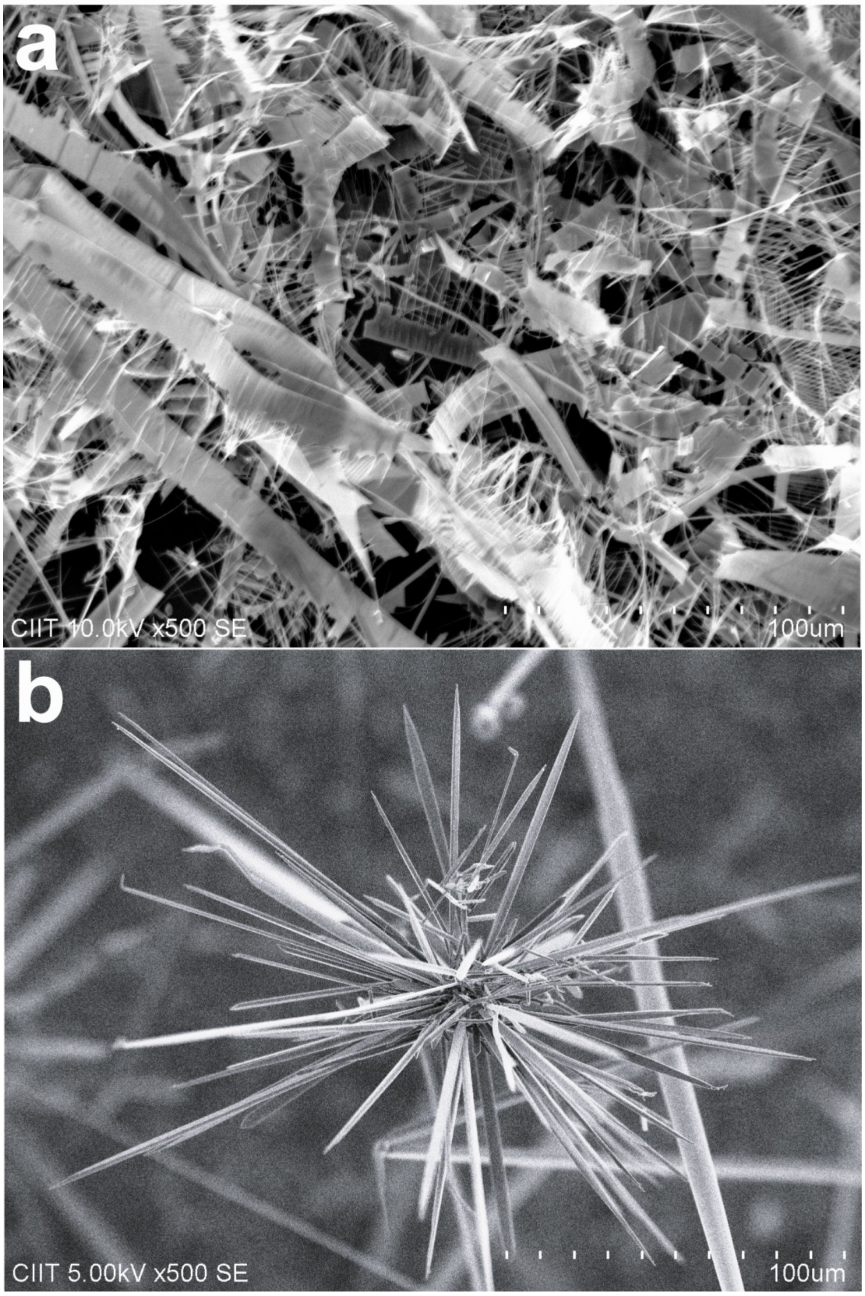

© 2012 by the authors; licensee MDPI, Basel, Switzerland. This article is an open access article distributed under the terms and conditions of the Creative Commons Attribution license (http://creativecommons.org/licenses/by/3.0/).
